# Supplementary material for: Factors Associated with Outpatient Satisfaction in Provincial Tertiary Hospitals in Nanchang, China: A Structural Equation Modeling Approach
Source: Int J Environ Res Public Health. 2022 Jul 6;19(14):8226. doi: 10.3390/ijerph19148226 (PMC9351663; doi:10.3390/ijerph19148226)
Supplement: Supplementary file 1 [file ijerph-19-08226-s001.zip › ijerph-1744845-supplementary.pdf]

S1. Basic Information

No.	Survey Contents
1	**Age:**________years old
2	**Gender:**<br>(1) Male (2) Female
3	**Education:**<br>(1) Postgraduate and above (2) Undergraduate / Junior college (3) Technical secondary school / Technical school (4) Senior high school<br>(5) Junior high school (6) Primary school and below
4	**Occupation:**<br>(1) Civil servant (2) State-owned enterprise employee (3) Private enterprise employee (4) Farmer (5) Freelancers (6) Retiree (7) Unemployed<br>(8) School student (9) Others__________
5	**Household Registration:**<br>(1) Rural (2) Urban
6	**Residence:**<br>(1) Local resident (2) Non-local resident
7	**Annual Household Income (yuan):**<br>(1) Less than 20,000 (2) 20,000~30,000 (3) 30,000~40,000 (4) 40,000~50,000 (5) 50,000~60,000<br>(6) 60,000~120,000 (7) 120,000~180,000 (8) More than 180,000
8	**Medical Insurance:**<br>(1) Free medicare (2) Urban employee basic medical insurance (3) Urban resident basic medical insurance<br>(4) New rural cooperative medical insurance (5) Commercial insurance (6) Medical aid (7) Without medical insurance<br>(8) Others__________

S2. Patient Satisfaction

No.	Indicators	Strongly Agree	Agree	Neutral	Disagree	Strongly Disagree	Not Experienced
1	I am satisfied with the waiting time for medical services.	5	4	3	2	1	0
2	I am satisfied with the hospital's patient guide services (e.g., guidance service, self-service equipment).	5	4	3	2	1	0
3	I am satisfied with the communication about my condition with my physician.	5	4	3	2	1	0
4	I am satisfied with the medical staff's explanation of medical examination reports.	5	4	3	2	1	0
5	I am satisfied with the medical staff's explanation of treatment protocol, medication methods, and precautions.	5	4	3	2	1	0
6	I do not think there were over-diagnosis and over-treatment during this visit.	5	4	3	2	1	0
7	I feel respect and comfort from the medical staff.	5	4	3	2	1	0
8	I am satisfied with the practice of protecting personal privacy during the consultation, treatment, and examination.	5	4	3	2	1	0
9	There are adequate seats in the waiting room.	5	4	3	2	1	0
10	There is clean water supplied in the waiting room.	5	4	3	2	1	0
11	The signage settings in the hospital are clear.	5	4	3	2	1	0
12	The spatial layout of the hospital is appropriate.	5	4	3	2	1	0
13	I think the medical fees for this visit bring a heavy financial burden on me.	5	4	3	2	1	0
14	Overall, I am satisfied with this visit.	5	4	3	2	1	0

Notes: The questionnaire was originally developed in Chinese. The English version presented here was translated only for the publication of this manuscript.
